# Changes in *Lolium perenne* transcriptome during cold acclimation in two genotypes adapted to different climatic conditions

**DOI:** 10.1186/s12870-015-0643-x

**Published:** 2015-10-17

**Authors:** Shamila Weerakoon Abeynayake, Stephen Byrne, Istvan Nagy, Kristina Jonavičienė, Thomas Povl Etzerodt, Birte Boelt, Torben Asp

**Affiliations:** Department of Agroecology – Crop Health, Aarhus University, Slagelse, Denmark; Department of Molecular Biology and Genetics, Science and Technology, Aarhus University, Slagelse, Denmark; Laboratory of Genetics and Physiology, Institute of Agriculture, Lithuanian Research Centre for Agriculture and Forestry, Kėdainiai distr, Lithuania

**Keywords:** Perennial ryegrass, Cold acclimation, Transcriptome, Fructan metabolism

## Abstract

**Background:**

Activation of numerous protective mechanisms during cold acclimation is important for the acquisition of freezing tolerance in perennial ryegrass (*Lolium perenne* L.). To elucidate the molecular mechanisms of cold acclimation in two genotypes (‘Veyo’ and ‘Falster’) of perennial ryegrass from distinct geographical origins, we performed transcriptome profiling during cold acclimation using RNA-Seq.

**Methods:**

We cold-acclimated plants from both genotypes in controlled conditions for a period of 17 days and isolated Total RNA at various time points for high throughput sequencing using Illumina technology. RNA-seq reads were aligned to genotype specific references to identify transcripts with significant changes in expression during cold acclimation.

**Results:**

The genes induced were involved in protective mechanisms such as cell response to abiotic stimulus, signal transduction, redox homeostasis, plasma membrane and cell wall modifications, and carbohydrate metabolism in both genotypes. ‘Falster’ genotype, adapted to cold climates, showed a stronger transcriptional differentiation during cold acclimation, and more differentially expressed transcripts related to stress, signal transduction, response to abiotic stimulus, and metabolic processes compared to ‘Veyo’. ‘Falster’ genotype also showed an induction of more transcripts with sequence homology to fructosyltransferase genes (*FTs*) and a higher fold induction of fructan in response to low-temperature stress. The circadian rhythm network was perturbed in the ‘Veyo’ genotype adapted to warmer climates.

**Conclusion:**

In this study, the differentially expressed genes during cold acclimation, potentially involved in numerous protective mechanisms, were identified in two genotypes of perennial ryegrass from distinct geographical origins. The observation that the circadian rhythm network was perturbed in ‘Veyo’ during cold acclimation may point to a low adaptability of ‘Veyo’ to low temperature stresses. This study also revealed the transcriptional mechanisms underlying carbon allocation towards fructan biosynthesis in perennial ryegrass.

**Electronic supplementary material:**

The online version of this article (doi:10.1186/s12870-015-0643-x) contains supplementary material, which is available to authorized users.

## Background

A period of low temperature stress (cold acclimation) can induce protective mechanisms, leading to morphological, physiological, and biochemical changes that are required for the acquisition of freezing tolerance in cold-tolerant plants [1]. These protective mechanisms include both alterations in gene expression, and metabolic re-adjustments. Previous studies have demonstrated the induction of genes involved in protective mechanisms such as cell redox homeostasis, signal transduction, cell wall and plasma membrane modifications, and metabolic re-adjustments during cold acclimation in a wide range of plant species [2–4]. Some cold regulated genes encode cryoprotectant proteins [5] and protect the cells from dehydration associated with freezing. Water-soluble carbohydrates (WSCs) also play protective roles during abiotic stresses. They can act as cryoprotectants [6], osmoprotectants [7] and also signaling molecules [8]. They might also play roles in neutralization of reactive oxygen species (ROS) [9] and membrane stabilization [10] during abiotic stresses. The likely scenario is that cold acclimation is a complex process with multiple components which are controlled by multiple regulatory mechanisms [11, 12].

The changes in the content and composition of WSCs during cold acclimation are associated with freezing tolerance in temperate grasses [13–15]. Perennial ryegrass (*Lolium perenne* L.), an agronomically important grass species, produces WSCs such as fructans and raffinose family oligosaccharides during cold acclimation [16, 17]. Some freezing-tolerant accessions of perennial ryegrass produce more WSCs during cold acclimation compared to freezing-susceptible accessions [18]. The water-soluble polymeric sugars, fructans, are the major reserve carbohydrates in perennial ryegrass. Many enzymes are involved in carbon allocation towards fructan biosynthesis. However, fructan structural diversity is mainly controlled by few fructosyltransferases (FTs) belonging to the family of glycoside hydrolases such as sucrose-sucrose 1-fructosyltransferase (1-SST) [19], fructan-fructan 1-fructosyltransferase (1-FFT) [20], sucrose-fructan 6-fructosyltransferase (6-SFT) [21], and fructan-fructan 6G-fructosyltransferase (6G-FFT) [22]. Other members of the same gene family, such as vacuolar invertases and cell wall invertases (CWIs) show high sequence similarity to FTs. Fructan exohydrolases (FEHs) such as 1-FEH and 6-FEH are involved in fructan degradation [23]. Both FTs and FEHs contribute to the quantitative and compositional changes of fructan during cold acclimation. Numerous transcription factors, protein kinases, and phosphatases are implicated in the regulation of genes involved in fructan biosynthesis [24–26].

In temperate grasses, a large proportion of the genome is cold-responsive [27, 28] as they are highly adaptive to the cold conditions. Induction of genes encode proteins such as cold-regulated, dehydration-responsive, and ice recrystallization inhibition proteins involved in protective mechanisms, has been shown in perennial ryegrass in response to low-temperature stress [28, 29]. However, stress response characteristics vary between different genotypes, especially between genotypes with very different geographic origins. Comparisons of transcriptomic data between such genotypes provide information about the plant adaptations to cold environments. We have recently demonstrated the improved cold stress tolerance and changes in fructan composition in ‘Veyo’ and ‘Falster’ genotypes of perennial ryegrass during cold acclimation [15]. ‘Falster’ is a Danish ecotype that is well adapted to cold climates, and ‘Veyo’ a Mediterranean variety well adapted to warmer climates [30]. ‘Falster’ showed a better adaptation during cold acclimation and faster recovery after freezing compared to ‘Veyo’ [15]. Furthermore, the genotypes differ in that ‘Falster’ must undergo a period of low temperature (vernalisation) in order to flower. ‘Veyo’ does not require a period of vernalisation to flower. A recent study has shown that both ‘Veyo’ and ‘Falster’ respond differently on a transcriptional level during vernalisation [31]. It would therefore be expected that ‘Veyo’ and ‘Falster’ would also respond differently on a transcriptional level during cold acclimation. Here we used these two types of perennial ryegrass to study the transcriptome profiles during cold acclimation using high throughput sequencing technologies and thereby gain a deeper insight into molecular mechanisms of cold acclimation. The specific aims of this study were: i) to identify candidate genes differentially expressed in perennial ryegrass during cold acclimation, ii) to identify molecular pathways differentiated between genotypes adapted to cold and warm climates, and iii) to identify the transcriptional mechanisms underlying carbon allocation towards fructan biosynthesis during cold acclimation.

## Results

### Differential expression of genes during cold acclimation

High-throughput RNA sequencing, generated ~2 Gb reads per sample. A total number of 157,264,629 reads of 50 bp were generated for the genotype ‘Veyo’ and a total of 151,608,297 reads were generated for ‘Falster’. In the ‘Veyo’ Trinity assembly, 50% of the total assembly was present in contigs of at least 1712 bp. In the ‘Falster’ Trinity assembly, 50 % of the total assembly was present in contigs of at least 1671 bp. The longest assembled contigs in ‘Veyo’ and ‘Falster’ had 15,228 bp and 15,362 bp, respectively. The average contig lengths in ‘Veyo’ and ‘Falster’ were 1078.60 bp and 1052.38 bp, respectively.

**Fig. 1:**
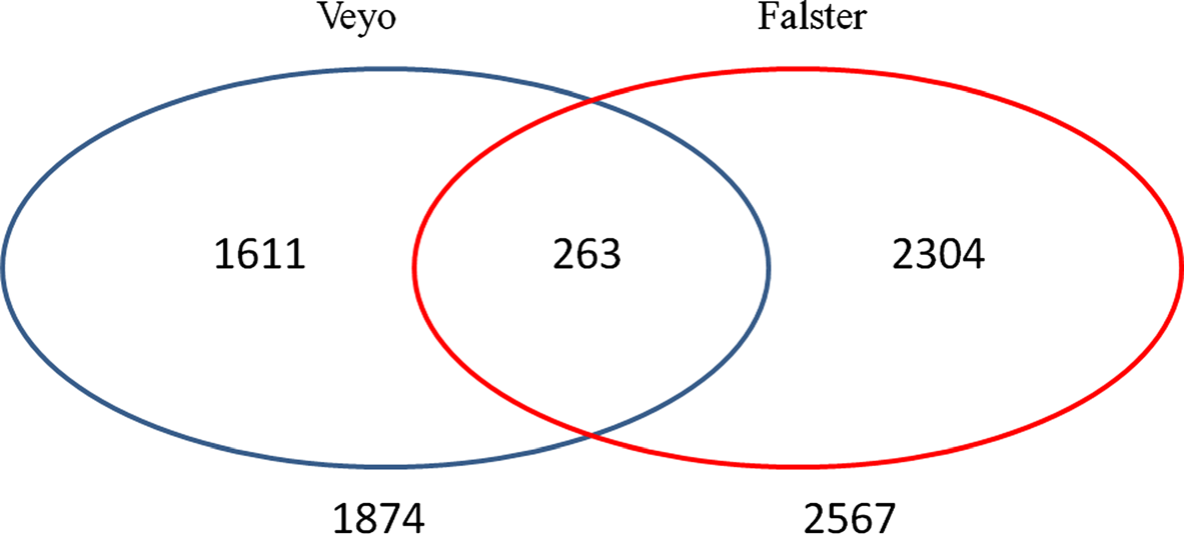
The number of differentially expressed transcripts common and specific for ‘Veyo’ and ‘Falster’ during cold acclimation

In total 1, 45,805 transcripts in ‘Veyo’ and 1, 44,062 transcripts in ‘Falster’ were identified. In a series of pairwise comparisons between the days, we identified 1874 differentially expressed transcripts in ‘Veyo’ and 2567 in ‘Falster’. There were 263 differentially expressed transcripts common for both ‘Veyo’ and ‘Falster’ (Fig. 1). Hierarchical cluster analysis performed using transcripts that were significantly differentially expressed (*P* ≤ 0.05) between any pairwise comparison (Additional file 1).

**Fig. 2:**
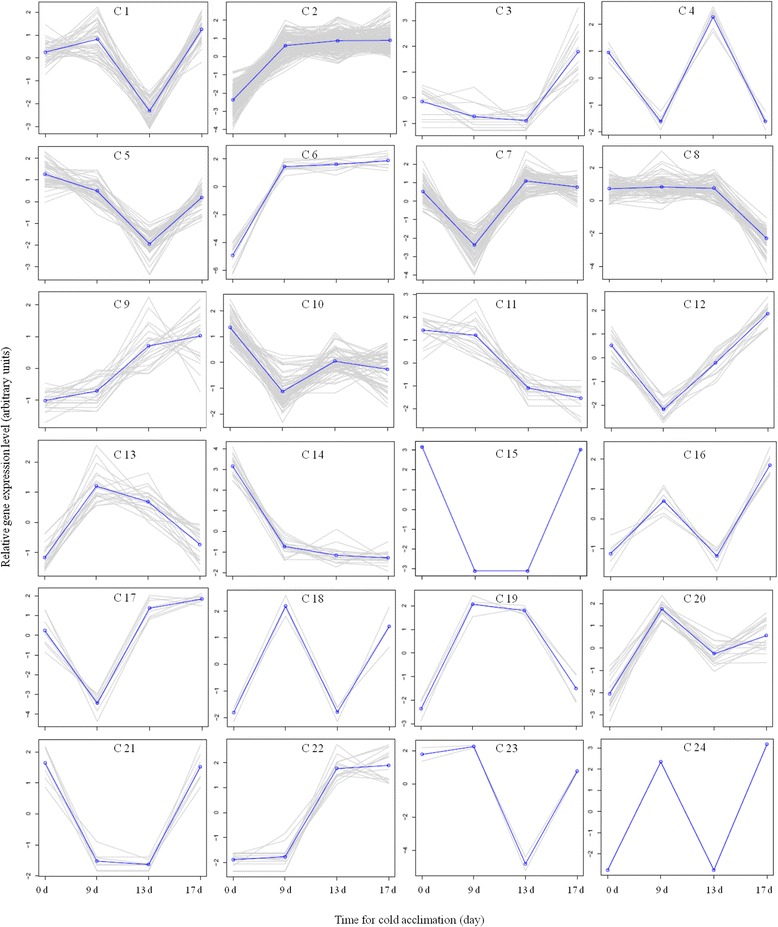
K-means clustering analysis of transcripts differentially expressed in ‘Veyo’ during cold acclimation. The x axis represents the sample collection time points (d) during cold acclimation. The y axis corresponds to the variance stabilized data from edgeR. Differentially expressed transcripts were divided into 24 categories (C 1 – C 24) according to their expression patterns. Plants were at 20 °C on the d 0 and at 7 °C on d 1 to d 17

**Fig. 3:**
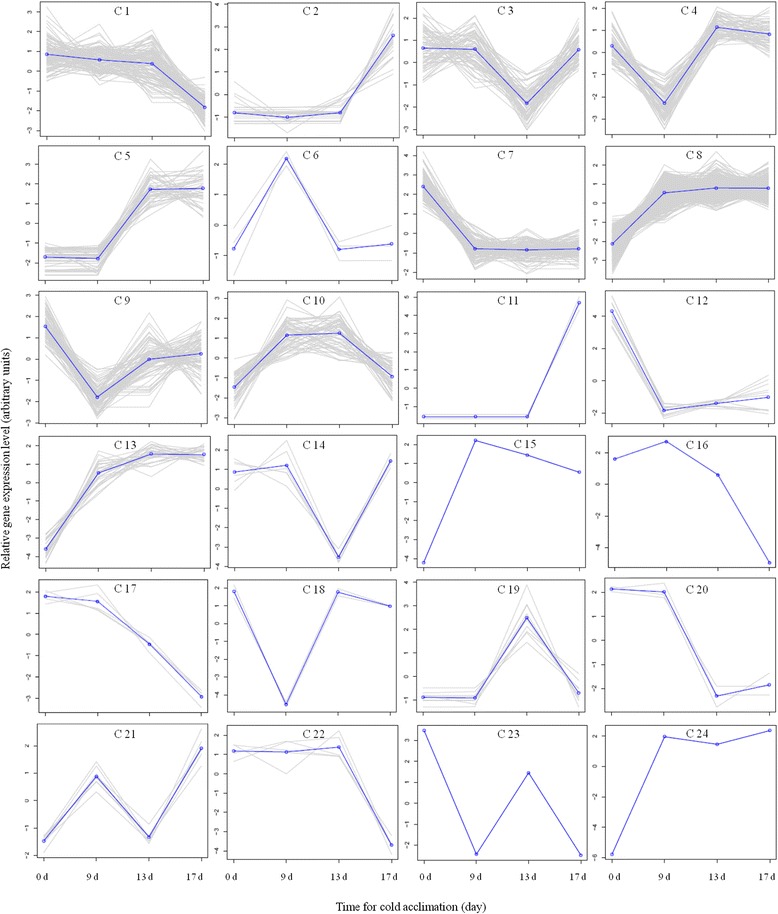
K-means clustering analysis of transcripts differentially expressed in ‘Falster’ during cold acclimation. The x axis represents the sample collection time points (d) during cold acclimation. The y axis corresponds to the variance stabilized data from edgeR. Differentially expressed transcripts were divided into 24 categories (C 1 – C 24) according to their expression patterns. Plants were at 20 °C on the d 0 and at 7 °C on d 1 to d 17

A total of 24 clusters of expression profiles were identified using K-means algorithm with distinguishable expression profiles during cold acclimation in each ‘Veyo’ or ‘Falster’ (Figs. 2 and 3, Additional files 2 and 3). The sudden drop of temperature from 20 °C to 7 °C lead to the rapid up- or down-regulation of transcripts in both ‘Veyo’ (Fig. 2, clusters C 2, C 6, C 10, and C 14) and ‘Falster’ (Fig. 3, clusters C 7, C 8, C 9, C 12, C 13, C 15, and C 24), indicative of transcripts involved in abiotic stress responses. The best BLAST hit descriptions of differentially expressed transcripts in ‘Veyo’ and ‘Falster’ are shown in Additional files 4 and 5, respectively. In general, there was an excellent correlation between the RNA-Seq results and the quantitative RT-PCR results (Additional file 6).

GO enrichment analysis using the Database for Annotation, Visualization, and Integrated Discovery (DAVID) tool [32] identified major biological, metabolic, and cellular processes significantly enriched (*P* ≤ 0.05) in ‘Veyo’ and ‘Falster’ during cold acclimation (Additional files 7, 8, 9, 10,11 and 12). Terms that were enriched in both ‘Veyo’ and ‘Falster’ were mainly related to the processes such as cell redox homeostasis that is important to maintain the redox environment of cells under stress conditions, signal transduction, response to abiotic stimulus, and carbohydrate metabolism. Analysis of terms under “cellular compartment” showed the enrichment of proteins associated with plasma membrane in “Veyo” and “Falster” (Additional files 11 and 12). These proteins included calcineurin B-like protein and elicitor-responsive protein, plasma membrane intrinsic protein aquaporins, cellulose synthase, ras-related protein RIC2, and secretory carrier-associated membrane protein. Terms such as extracellular region, chloroplast, golgi apparatus, and cell-wall were also found to be significantly enriched in both ‘Veyo’ and ‘Falster’. The DAVID analysis also showed the protein domains that were significantly enriched (*P*<0.05) in the differentially expressed transcripts (Additional files 13 and 14). Enriched SMART domains revealed the presence of functional domains such as serine-threonine protein kinase catalytic (S_TKc), serine-threonine phosphatases, family 2C, catalytic (PP2Cc), heat shock factor (HSF), cyclin, and formin homology (FH) domains in both genotypes.

### Divergence of gene expression-profiles between “Veyo” and “Falster” genotypes during cold acclimation

**Fig. 4:**
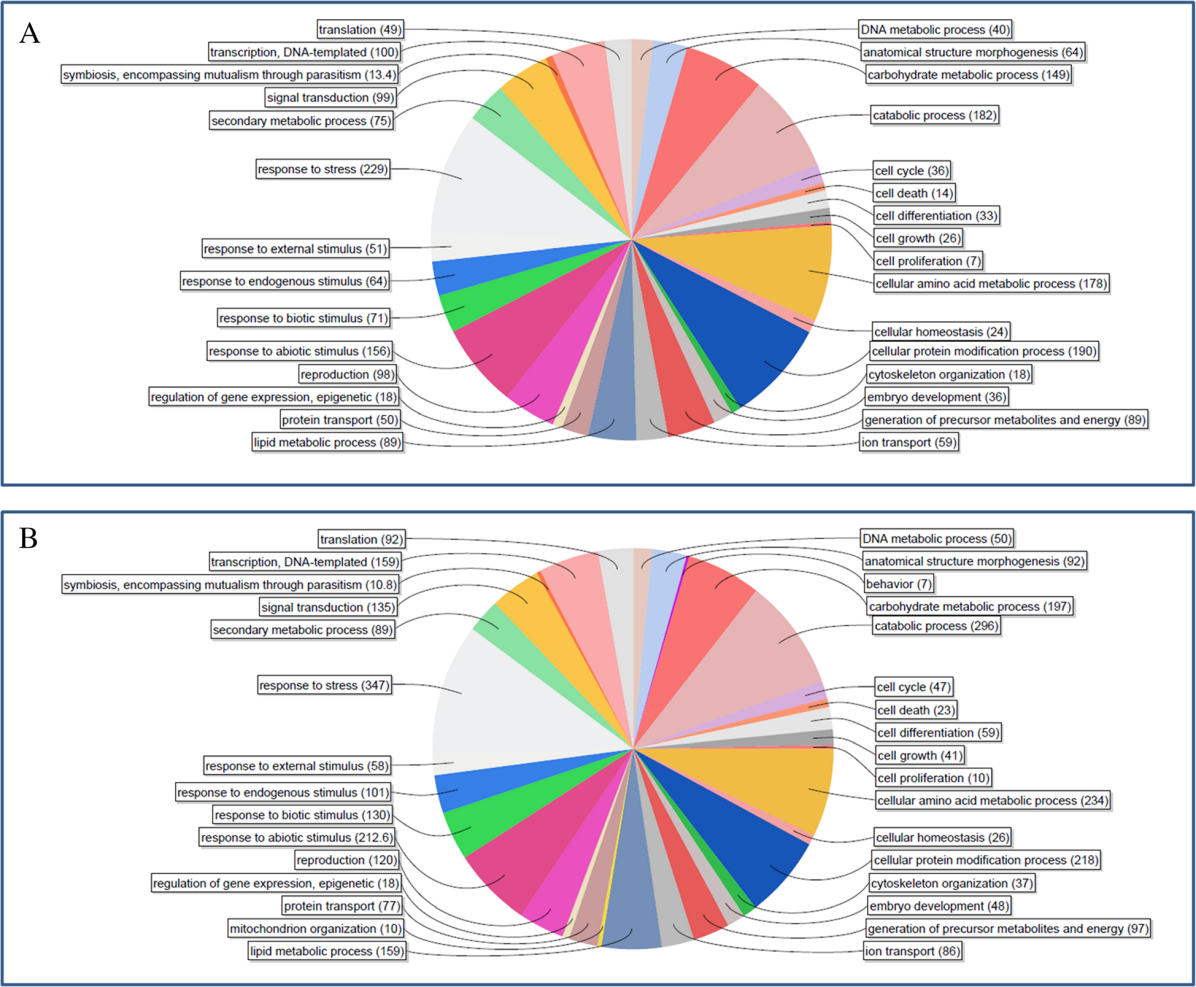
The pie charts of the biological processes of unigenes differently expressed during cold acclimation. The pie charts of the biological processes (GO level 4) of unigenes differently expressed in “Veyo” (**a**) and “Falster” (**b**) genotypes during cold acclimation. The colours depict distinct categories. Number of transcripts associated to each category is shown in brackets

Our results show more differentially expressed transcripts in ‘Falster’ compared to ‘Veyo’ during cold acclimation. Gene Ontology annotation (GO) of the possible functions of unigenes using Blast2GO showed transcripts related to stress, signal transduction, response to abiotic stimulus, and metabolic processes such as lipid, amino acid, and carbohydrates (Fig. 4). ‘Falster’ showed more transcripts in each of these categories compared to ‘Veyo’. Analysis of protein domains using DAVID tool revealed the enrichment of EF-hand, calcium binding domain only in ‘Falster’ (Additional files 14).

**Fig. 5:**
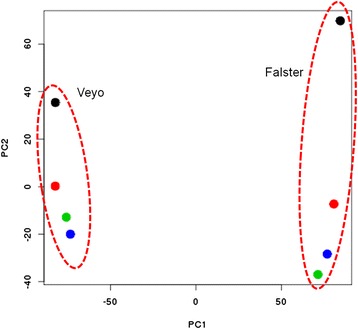
The principle component analysis (PCA) of differently expressed transcripts during cold acclimation. PCA of differently expressed transcripts between ‘Veyo’ and ‘Falster’ genotypes and between lengths of time in days that plants were exposed to cold acclimation are shown. PC1 separates samples according to genotype and explains 66 % of variance. PC2 separates samples according to the length of time in d that plants were exposed to cold acclimation and explains 12 % of the variance. Black, red, green and blue dots represent 0, 9, 13 and 17 d of cold acclimation

In order to make comparisons between the expression profiles of ‘Veyo’ and ‘Falster’, we used common reference *L. perenne* transcriptome [33] for differential analysis. Principle component analysis (PCA) showed that the transcription profiles were separated according to genotype on PC1, which explained 66 % of the variance (Fig. 5). The second PC separated samples according to treatment and explained 12 % of the variance. There was greater separation on PC2 for samples from the ‘Falster’ genotype, indicating a stronger transcriptional differentiation during cold acclimation.

There were two KEGG pathways that were significantly enriched in the ‘Veyo’ differentially expressed gene set; these were ‘Phenylpropanoid biosynthesis’ (*P* = 2.80E-02) and ‘Circadian rhythm’ (*P* = 2.90E-02). Down regulation of genes encode enzymes involved in phenylpropanoid biosynthesis such as phenylalanine ammonia-lyase, 4-coumarate--CoA ligase, and O-methyl transferase, and also the down regulation of genes encode two-component response regulators involved in circadian rhythm [34], in response to the sudden drop of temperature from 20 °C to 7 °C, was observed in ‘Veyo’ (Fig. 2, cluster C 10). Only a single KEGG pathway was significantly enriched in the ‘Falster’ differentially expressed gene set; this was ‘Proteasome’ (*P* = 2.00E-03). The genes encode proteasome regulatory subunit proteins up regulated in ‘Falster’ during cold acclimation (Fig. 3, cluster C 8). Analysis of terms under “biological processes” showed that terms such as rhythmic process (*P* = 6.30E-03), two-component signal transduction system (*P* = 2.60E-02), nuclear division (*P* = 3.30E-02), and mitosis (*P* = 3.30E-02) were only enriched in ‘Veyo’ (Additional file 7).

### Induction of genes involved in fructan metabolism and carbon allocation towards fructan biosynthesis during cold acclimation

Among the differentially expressed transcripts, 345 transcripts were related to carbohydrate metabolism or carbohydrate binding (Additional files 4 and 5). Transcripts potentially involved in fructan biosynthesis were classified into four groups, based on their deduced amino acid sequence similarity to the previously characterized genes. These four groups are FTs, FEHs, invertases, and other genes potentially involved in carbon flux diversion towards fructan biosynthesis (Additional file 15).

Seven transcripts from ‘Falster’ that were up regulated (Fig. 3, clusters C 8 and C 13), showed deduced amino acid sequence similarity with the functionally characterized Lp1-SST [20], Lp6-SFT, and Lp6G-FFT [22], of which five showed over 90 % deduced amino acid sequence similarity over an alignment of 500 amino acids (Additional file 15). Three transcripts from ‘Veyo’ (Fig. 2, cluster C 14), and four transcripts from ‘Falster’ (Fig. 3, clusters C 3 and C 7) that were down regulated, showed deduced amino acid sequence similarity with FT-like protein (LpFTL) [35]. Seven transcripts from ‘Veyo’ were classified as invertases, of which two were CWIs. One transcript from ‘Veyo’ (Fig. 2, cluster C 14) and two transcripts from ‘Falster’ (Fig. 3, cluster C 7) classified as soluble acid invertases, were down regulated during cold acclimation. Two transcripts from ‘Falster’ which were down regulated (Fig. 3, cluster C 7), showed over 88 % deduced amino acid sequence similarity with 6-FEH from Timothy (*Phleum pratense*) [36]. Another transcript from ‘Falster’ that was up regulated (Fig. 3, cluster C 8) showed 99 % sequence similarity with Lp6-FEH.

**Fig. 6:**
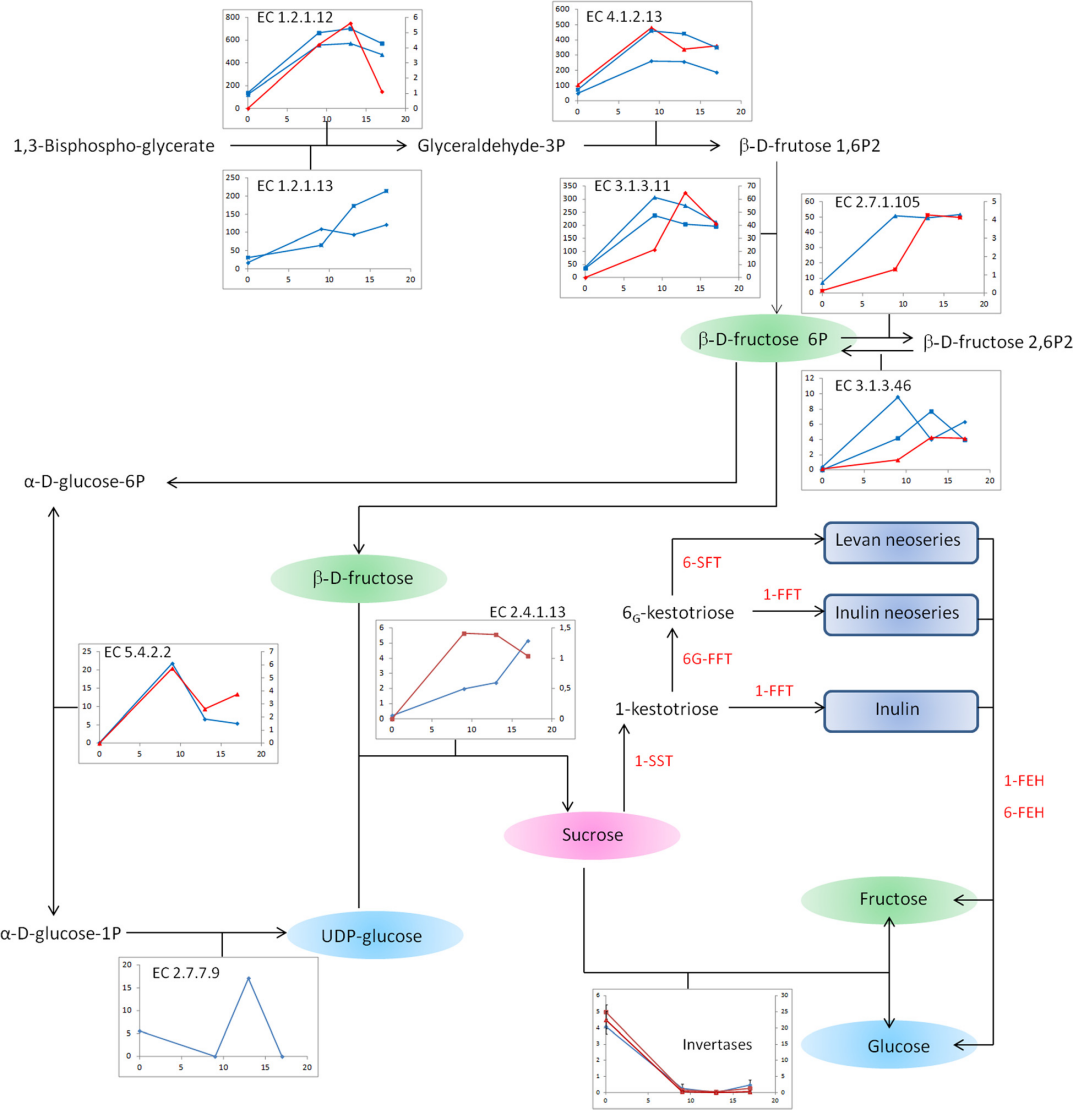
A model for carbon flux diversion towards fructan biosynthesis in perennial ryegrass. The model is based on the unigenes related to KEGG pathways. Enzymes involved in fructan metabolism: sucrose-sucrose 1-fructosyltransferase (1-SST), fructan-fructan 1-fructosyltransferase (1-FFT), fructan-fructan 6G-fructosyltransferase (6G-FFT), sucrose-fructan 6-fructosyltransferase (6-SFT), and fructan exohydrolases (1-FEH and 6-FEH). KEGG enzyme data base entry numbers (EC) of other enzymes involved in carbon flux diversion towards fructan biosynthesis: glyceraldehyde-3-phosphate dehydrogenase (GAPDH) (EC 1.2.1.12), GAPDH (NADP+) (EC 1.2.1.13), fructose-bisphosphate aldolase (EC 4.1.2.13), fructose-bisphosphatase (EC 3.1.3.11), fructose-6-phosphate,2-kinase (EC 2.7.1.105), fructose-2,6-bisphosphatase (EC 3.1.3.46), sucrose synthase (EC 2.4.1.13), UDP-glucose pyrophosphorylase (EC 2.7.7.9) and glucose phosphomutase (EC 5.4.2.2). Metabolic intermediates: glyceraldehyde-3-phosphate (Glyceraldehyde-3P), β-D-frutose 1,6-bisphosphate (β-D-frutose 1,6P2), β-D-fructose 6-phosphate (β-D-fructose 6P), and β-D-fructose 2,6-bisphosphate (β-D-fructose 2,6P2). The expression patterns of the transcripts from perennial ryegrass variety ‘Veyo’ (blue) and ecotype ‘Falster’ (red) showing deduced amino acid sequence similarity with KEGG enzymes are shown. Expression profiles are only shown in cases where significant differential expression was identified during cold acclimation. The x-axis represents time during cold acclimation (d) and y-axis represents relative gene expression (arbitrary units)

**Fig. 7:**
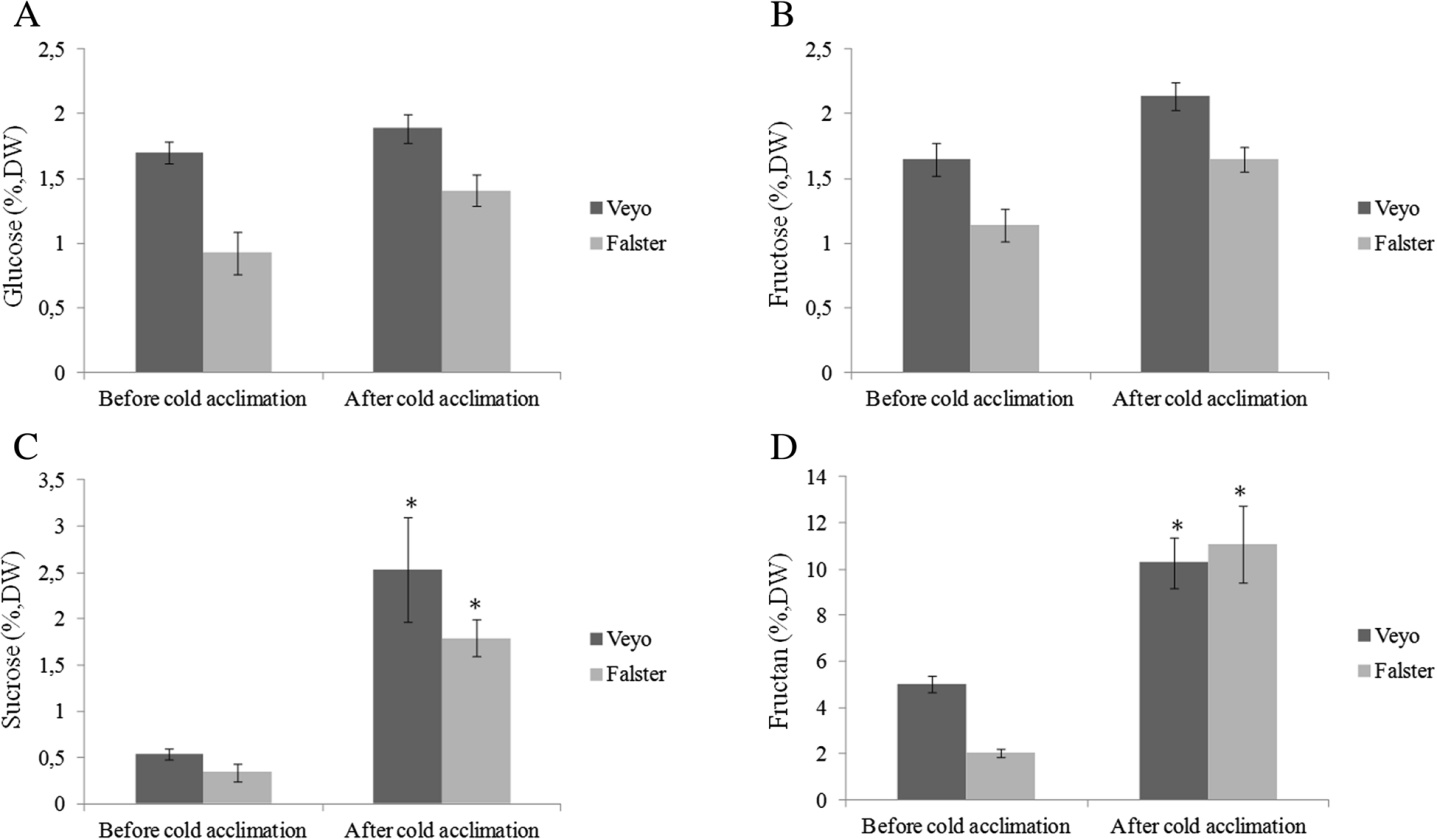
Quantitative changes in water soluble carbohydrates (WSCs) ‘Veyo’ and ‘Falster’ genotypes of perennial ryegrass. The changes (%, DW) in glucose (**a**), fructose (**b**), sucrose (**c**), and fructan (**d**) contents before cold acclimation when the plants at 20 °C, and after 17 days at 7 °C are shown. Data represent the mean ± SE, obtained from three replicates of the analysis. Asterisks indicate significant differences (*P* < 0.05) compared to before cold acclimation

A model for carbon flux diversion towards fructan biosynthesis in perennial ryegrass is shown in Fig. 6. The differentially expressed transcripts during cold acclimation potentially involved in carbon flux diversion towards fructan biosynthesis showed deduced amino acid sequence similarity to proteins such as glyceraldehyde-3-phosphate dehydrogenase (GAPDH) (EC 1.2.1.12), GAPDH (NADP^+^) (EC 1.2.1.13), fructose-bisphosphate aldolase (EC 4.1.2.13), fructose-bisphosphatase (EC 3.1.3.11), fructose-6-phosphate,2-kinase (EC 2.7.1.105), fructose-2,6-bisphosphatase (EC 3.1.3.46), sucrose synthase (EC 2.4.1.13), UDP-glucose pyrophosphorylase (UGPase) (EC 2.7.7.9), and glucose phosphomutase (EC 5.4.2.2). Quantitative changes in glucose, fructose, sucrose and fructan are shown in Fig. 7. The fructan content was increased around two-fold in ‘Veyo’ and around five-fold in ‘Falster’ during cold acclimation. Sucrose content was increased around five-fold in both genotypes.

## Discussion

### Activation of protective mechanisms during cold acclimation

Transcriptome analysis showed the activation of numerous protective mechanisms in ‘Veyo’ and ‘Falster’ during cold acclimation. Moreover, a number of processes enriched in the sets of genes differentially expressed during cold acclimation were common to both ‘Veyo’ and ‘Falster’. This is not surprising because both genotypes have shown acquisition of freezing tolerance during cold acclimation [15]. Consistent with the previous studies [2, 4, 37] our results also showed the induction genes involved in protective mechanisms such as redox homeostasis mechanisms, signal transduction, cell membrane stabilization, and carbohydrate metabolism during cold acclimation. Induction of cold-responsive genes such as *cold-regulated*, *dehydration-responsive*, *late-embryogenesis-abundant*, and *ice recrystallization inhibition* homolog genes has been shown previously in perennial ryegrass cv. ‘Caddyshack’ during cold acclimation [28]. Our results showed the induction of these genes also in ‘Veyo’ and ‘Falster’ genotypes during cold acclimation. Analysis of differentially expressed transcripts during cold acclimation revealed that up- or down-regulation of large number of transcripts in both ‘Veyo’ and ‘Falster’. Down regulation of genes involved in mechanisms such as photosynthesis and respiration during cold acclimation, has been previously shown [28, 38]. Our results also support the hypothesis that in addition to gene induction, gene repression might also play roles in cold acclimation [4].

Induction of genes encode plasma membrane associated proteins such as calcineurin B-like protein, and elicitor-responsive protein might play important roles in Ca^2+^-sensing and defense signaling [39, 40] in both genotypes. The secretory carrier-associated membrane protein (SCAMP6) protein enriched in “Veyo” and “Falster” might be involved in the membrane trafficking of polysaccharides. The co-localization of NtSCAMP2 from *Nicotiana tabacum* and pectin in vesicular compartments has been shown [41], suggesting that NtSCAMP2 plays a role in intracellular vesicular-mediated transportation of polysaccharides. Induction of genes encode plasma membrane intrinsic protein aquaporins has been observed in both genotypes. These proteins might be involved in cellular hydraulic conductivity, transportation of solutes across membranes, and carbohydrate compartmentation [42, 43] during cold acclimation. Enrichment of functional domains such as S_TKc, PP2Cc, HSF, cyclin, and FH during cold acclimation indicates the activation of processes such as phosphorylation and dephosphorylation of target enzymes, protein folding and protein translocation, cytoprotection, transcriptional regulation, and cytoskeleton rearrangements [44–47].

‘Falster’ genotype, adapted to cold climates showed a stronger transcriptional differentiation during cold acclimation suggestive of differences in the cold acclimation responses between these two perennial ryegrass genotypes adapted to different climatic conditions. Previous studies have demonstrated coordinated expression of functionally diverse *FTs* and the association of specific isoforms with the high sugar trait in perennial ryegrass [20, 48]. Our results show the induction of more transcripts with sequence homology to fructosyltransferase genes (*FTs*) in the ‘Falster’ genotype adapted to cold climates, compared to ‘Veyo’ in response to low-temperature stress. Some of them might represent different isoforms of *FTs*. Induction of specific and / or multiple isoforms of *FTs* might play roles in higher fold induction of fructan in cold adapted genotypes of perennial ryegrass.

The KEGG pathways ‘Phenylpropanoid biosynthesis’ and ‘Circadian rhythm’ were significantly enriched in the differentially expressed gene set of ‘Veyo’ originating from Italy. Phenylpropanoid metabolism produces a large array of secondary metabolites that are involved in protective mechanisms against abiotic stresses [49, 50]. The observation that the down regulation of genes involved in these pathways may point to a low adaptability of this Italian variety to low temperature stresses. Our results indicate the up regulation of proteasome pathway in ‘Falster’ during cold acclimation. This may point to a high adaptability of ‘Falster’ to low temperature stresses. The proteasome pathway degrades misfolded proteins that are generated in response to environmental stresses [51].

Apart from the induction of genes involved in numerous protective mechanisms, post-transcriptional and post-translational mechanisms regulating the function of proteins [52] also play roles in abiotic stress tolerance. Our results showed divergence of gene expression-profiles between “Veyo” and “Falster” genotypes during cold acclimation. The proteomic response to cold acclimation can be also varied between perennial ryegrass genotypes [53]. Further investigation of such differences between genotypes is important for a better understanding of biological processes involved in cold acclimation.

### Coordinated gene expression directs carbon flux towards fructan biosynthesis

It has already been suggested that fructans play important roles in abiotic stress tolerance in temperate grasses [13, 15]. Therefore, in this study we looked at the expression of genes involved in fructan metabolism. Genes encoding transcription factors such as MYB, bZIP, AP2/EREBP, WRKY, and NAC induced in ‘Veyo’ and ‘Falster’ during cold acclimation might play important roles in coordinating genes involved in protective mechanisms [54–56]. Previous studies have shown the up-regulation of *1-SST* and *6-SFT* promoter-driven reporter genes by a R2R3 MYB transcription factor from wheat [57], suggesting that transcription factors might play important roles in coordinating fructan-related gene expression. Consistent with the previous studies [28, 31], our results also show the induction of genes encoding transcription factors potentially involved in coordination of fructan-related gene expression in perennial ryegrass during cold acclimation. A large number of genes encoding kinases and phosphatases were also differentially expressed during cold acclimation in both ‘Veyo’ and ‘Falster’ (Additional files 4 and 5). Some kinases and phosphatases are involved in the sucrose-mediated induction of fructan synthesis in plants [25, 26].

During cold acclimation, carbon fixation by photosynthesis exceeds the demand for growth and development [58]. Therefore, carbon flux is directed towards fructan biosynthesis. In this study, proteins involved in carbon fixation, such as GAPDH, fructose-bisphosphate aldolase, and fructose-bisphosphatase, were induced during cold acclimation, and these may be involved in directing flux towards β-D-fructose 6-phosphate (β-D-Fru 6P) synthesis. In addition, the induction of genes involved in fructose and mannose metabolism was observed, that also direct the carbon flux towards β-D-Fru 6P. Fructose-6-phosphate,2-kinase (EC 2.7.1.105) and fructose-2,6-bisphosphatase (EC 3.1.3.46) enzymes catalyse the reversible interconversion between β-D-Fru 6P and β-D-Fructose 2,6-bisphosphate (β-D-Fru 2,6P2). As a regulatory molecule, β-D-Fru 2,6P2 affects photosynthetic carbon partitioning in *Arabidopsis* [59]. Previous studies have shown that metabolic intermediates become available for sucrose and fructose synthesis when the level of β-D-Fru 2,6P2 decreases [60]. Interconversion of D-Fru 6P and D-glucose-6-phosphate can be carried out by glucose-6-phosphate isomerase activity. D-glucose-6-phosphate can be converted into UDP-glucose by glucose-phosphomutase (EC 5.4.2.2) and UGPase (EC 2.7.7.9) enzyme activities. In our study, induction of glucose-phosphomutase was observed in both ‘Veyo’ and ‘Falster’. However, induction of UGPase was observed only in ‘Veyo’ during cold acclimation, whilst the induction of sucrose synthase (EC 2.4.1.13), the enzyme involved in sucrose synthesis by catalysing the reaction between UDP-glucose and D-fructose, was observed in both ‘Veyo’ and ‘Falster’. In addition, carbon flux diversion towards sucrose synthesis increases substrates for FTs to produce inulin, inulin neoseries, and levan neoseries fructans in perennial ryegrass (Fig. 6).

## Conclusions

RNA-Seq enabled an untargeted profiling of the transcriptome of two perennial ryegrass genotypes adapted to different climatic conditions. The stronger transcriptional differentiation during cold acclimation in genotype ‘Falster’ explains its higher adaptability to cold climates compared to ‘Veyo’. This study also describes the differentially expressed genes involved in fructan metabolism and other protective mechanisms during cold acclimation that can be useful for further research such as the functional analysis of genes, and comparative genomic studies.

## Methods

### Plant materials and growth conditions

Perennial ryegrass (*Lolium perenne* L.) variety ‘Veyo’ originating from Italy and adapted to warmer climates, and ecotype ‘Falster’ originating from Denmark and adapted to cold climates [30] were propagated vegetatively in a glasshouse. Following propagation, the plants were grown in a glasshouse (photoperiod of 9 h:15 h, light:dark) at ~20 °C for 12 weeks. The plants were cut (12 cm above the soil level) two times during the 12 weeks in glasshouse, and another time prior to transfer into a controlled environment chamber. The plants (at the pre-elongation stage) were then transferred to a growth chamber for 7 d (photoperiod of 9 h:15 h, light:dark), with 450 μmol photons m^−2^ s^−1^ light intensity at 20 °C. The plants were cold-acclimated in a controlled growth room for 17 d (photoperiod of 9 h:15 h, light:dark), with 450 μmol photons m^−2^ s^−1^ light intensity and relative humidity of ~70 % at 7 °C as previously described [15].

### Measurement of total fructan

The blades of fully developed leaves harvested in three biological replicates before and after cold acclimation were freeze-dried and ground to powder. Soluble carbohydrates were extracted from 100 mg of sample with 25 ml of 0.1 M acetate buffer (pH 5.0) for 1 h at 65 °C. Extracts from each sample (2 ml) were hydrolysed using an equal volume of 0.074 M H_2_SO_4_ for 70 min at 80 °C. The amounts of glucose and fructose before and after acid hydrolysis were measured, using the hexokinase–phosphoglucose isomerase glucose-6-phosphate dehydrogenase system, by calculating the sucrose and fructan contents, as previously described [13].

### RNA-Seq analysis

We have previously demonstrated an improved cold stress tolerance in both ‘Veyo’ and ‘Falster’ during the first 17 d of cold acclimation [15]. Therefore, in this study, same time points were selected for RNA-Seq analysis to elucidate the molecular mechanisms of cold acclimation. The blades of fully developed leaves were harvested in three biological replicates at the onset of the daily photoperiod on d 0, 9, 13, and 17 of cold acclimation at 7 °C. The samples were frozen in liquid nitrogen immediately after harvesting and stored at –80 °C. until the analysis of carbohydrates and gene expression levels was performed. The samples were ground separately in liquid nitrogen. Total RNA was extracted using RNeasy® Plant Mini Kit (Qiagen), and On-column DNAse I digestion (Qiagen) was performed to avoid genomic DNA contamination, according to the manufacturer’s instructions. Total RNA quality was verified using an Agilent 2100 Bioanalyzer (RNA 6000 Nano Assay) and quantified by Quant-iT^TM^ RiboGreen® RNA Reagent assay, according to the manufacturer’s protocol. Total RNA samples were prepared for sequencing, using the TruSeq RNA Sample Preparation Kit (Illumina).

Illumina HiSeq 2000 platform was used for high-throughput sequencing (Beijing Genomics Institute, Hong Kong). Where the average quality score in a sliding window analysis (10 % of read length) fell below 20, poor-quality bases towards the 3′ end of reads were trimmed using Sickle [61]. Reads shorter than 40 bp after trimming were discarded. *De novo* assembly of RNA-Seq data was carried out using the Trinity assembler, as previously described [62]. *De novo* transcriptomes were assembled for ‘Veyo’, and for ‘Falster’ separately (minimum contig length of 200, and minimum kmer coverage of 2).

RNA-Seq Reads were aligned back to their respective assemblies using Bowtie as previously described [63]. The abundance estimates of transcripts separately for each time point (d 0, 9, 13, and 17 of the cold acclimation) and genotype (‘Veyo’ and ‘Falster’) were calculated using RSEM [64]. Pairwise comparisons were carried out between all the selected time points and differentially expressed transcripts (*P* ≤ 0.05) were identified using edgeR as previously described [65].

RNA-Seq count data from each time point was normalized using trimmed mean of M-values (TMM) normalization [66] in edgeR to compute fragments per feature kilobase per million read mapped (FPKM) values. These variance stabilized data was used for clustering the transcripts. The transcripts showing differential expression at any time point during cold acclimation were clustered using K-means algorithm. Hierarchical cluster analysis was carried out separately on two sets of differentially expressed transcripts, a differentially expressed set from ‘Veyo’, and a differentially expressed set from ‘Falster’. Each set contained transcripts that were significantly differentially expressed (*P* ≤ 0.05) between any paired comparison for samples from ‘Veyo’ or ‘Falster’. The differentially expressed gene sets were mapped onto Kyoto Encyclopedia of Genes and Genomes (KEGG) pathway maps to identify networks differentially regulated between ‘Veyo’ and ‘Falster’.

The differentially expressed transcripts were annotated using Blast2GO as previously described [31]. Functional annotation information was also assigned to each transcript by using each sequence as a query in a BLASTx against a UniProt database. Gene ontology annotation (GO) of the possible functions of unigenes was carried out using the Database for Annotation, Visualization, and Integrated Discovery (DAVID) bioinformatic tool [32]. In order to compare the differentially expressed transcript sets identified in ‘Veyo’ and ‘Falster’, BLASTn (E-value threshold 10^e-10^) was used to map the transcripts back to a common reference *L. perenne* transcriptome [33] as previously described [31]. Principle component analysis (PCA) plot was generated based on variance stabilized expression data in order to separate transcription profiles according to genotype and treatment.

The RNA-Seq results were validated using quantitative reverse transcriptase-polymerase chain reaction (qRT-PCR) standard curve method for absolute quantification on a selection of genes potentially involved in fructan metabolism that were identified as differentially expressed during cold acclimation. PCR procedure and the primers used for the expression analysis of *Lp1-SST*, *Lp1-FFT*, *Lp6G-FFT*, and *Lp1-FEH* genes have been previously described [15]. PCR primers and the quantitative RT-PCR primers used for the expression analysis of *LpFTL*, *LpCWI-1* and *LpCWI-2* genes are shown (Additional files 16 and 17).

### Availability of supporting data

Illumina sequence data are available from ArrayExpress with the accession number E-MTAB-2779.

## Additional files

Additional file 1:
**Heat maps derived from hierarchical clustering of transcripts differentially expressed in ‘Veyo’ and ‘Falster’ during cold acclimation.** Expression patterns across d 0, 9, 13, and 17 of cold acclimation in (A) ‘Veyo’ and (B) ‘Falster’. Green represents up-regulation, and red represents down-regulation. (TIFF 494 kb) Additional file 2:
**The transcripts classified into different clusters based on their expression patterns using K-means clustering analysis of transcripts differentially expressed in ‘Veyo’ during cold acclimation.** (XLSX 18 kb) Additional file 3:
**The transcripts classified into different clusters based on their expression patterns using K-means clustering analysis of transcripts differentially expressed in ‘Falster’ during cold acclimation.** (XLSX 31 kb) Additional file 4:
**Differentially expressed transcripts in ‘Veyo’ genotype of perennial ryegrass (** ***Lolium perenne*** **L.) during cold acclimation.** (XLSX 289 kb) Additional file 5:
**Differentially expressed transcripts in ‘Falster’ genotype of perennial ryegrass (** ***Lolium perenne*** **L.) during cold acclimation.** (XLSX 375 kb) Additional file 6:
**Validation of RNA-Seq results using quantitative RT-PCR.** The expression analysis of selected fructan-related genes differentially expressed during cold acclimation in perennial ryegrass are shown. Plants were at 20 °C on the d 0 and at 7 °C on the d 9, 13 and 17. The normalized transcript levels obtained in the RNA-Seq experiment (lines, right scale) and quantitative RT-PCR results (solid bars, left scale) are shown. Expression of (A) NGB_70583_c0_seq6, (B) NGB_67546_c0_seq4, and (C) NGB_52509_c0_seq1 transcripts from ecotype ‘Falster’, homologues to *FTs.* Expression of (D) NGB_56538_c0_seq2, and (E) NGB_43481_c0_seq3 transcripts from ecotype ‘Falster’, homologues to *Lp-FEH* and *LpFTL* genes respectively. Expression of (F) Veyo_57878_c1_seq2, (G) Veyo_65000_c0_seq1, and (H) Veyo_72656_c0_seq1 transcripts from variety ‘Veyo’, homologues to *LpFTL*, *LpCWI-1* and *LpCWI-2* genes respectively. Quantitative RT-PCR data were normalized by geometric averaging of elongation factor 1-alpha (*LpEF1a*), actin (*LpACT11*) and eukaryotic initiation factor 4A (*LpeIF4a*) internal control genes. Data represent mean ± SE obtained from three biological replicates of the analysis. (TIFF 439 kb) Additional file 7:
**Major biological processes of differentially expressed transcripts in ‘Veyo’ genotype of perennial ryegrass (** ***Lolium perenne*** **L.) during cold acclimation identified by the DAVID tool.** (XLSX 15 kb) Additional file 8:
**Major biological processes of differentially expressed transcripts in ‘Falster’ genotype of perennial ryegrass (** ***Lolium perenne*** **L.) during cold acclimation identified by the DAVID tool.** (XLSX 14 kb) Additional file 9:
**Major molecular functions of differentially expressed transcripts in ‘Veyo’ genotype of perennial ryegrass (** ***Lolium perenne*** **L.) during cold acclimation identified by the DAVID tool.** (XLSX 12 kb) Additional file 10:
**Major molecular functions of differentially expressed transcripts in ‘Falster’ genotype of perennial ryegrass (** ***Lolium perenne*** **L.) during cold acclimation identified by the DAVID tool.** (XLSX 11 kb) Additional file 11:
**Major cellular compartments of differentially expressed transcripts in ‘Veyo’ genotype of perennial ryegrass (** ***Lolium perenne*** **L.) during cold acclimation identified by the DAVID tool.** (XLSX 11 kb) Additional file 12:
**Major cellular compartments of differentially expressed transcripts in ‘Falster’ genotype of perennial ryegrass (** ***Lolium perenne*** **L.) during cold acclimation identified by the DAVID tool.** (XLSX 12 kb) Additional file 13:
**Major functional domains of differentially expressed proteins in ‘Veyo’ genotype of perennial ryegrass (** ***Lolium perenne*** **L.) during cold acclimation identified by the DAVID tool.** (XLSX 10 kb) Additional file 14:
**Major functional domains of differentially expressed proteins in ‘Falster’ genotype of perennial ryegrass (** ***Lolium perenne*** **L.) during cold acclimation identified by the DAVID tool.** (XLSX 10 kb) Additional file 15:
**Differentially expressed fructan-related transcripts and other transcripts potentially involved in carbon flux diversion towards fructan biosynthesis during cold acclimation in perennial ryegrass (** ***Lolium perenne*** **L.).** (XLSX 20 kb) Additional file 16:
**PCR primers used to amplify the template DNA for absolute quantification of gene expression using quantitative RT-PCR.** Forward and reverse primers used to amplify the template DNA for the expression analysis of the genes encode fructosyltransferase-like (LpFTL), and cell wall invertases (LpCWI-1 and LpCWI-2). (DOCX 20 kb) Additional file 17:
**Gene-specific primers used for quantitative RT-PCR analysis.** Forward and reverse gene specific primers used for the expression analysis of the genes encode fructosyltransferase-like (LpFTL) and cell wall invertases (LpCWI-1 and LpCWI-2). (DOCX 20 kb)

## Abbreviations

FTs: Fructosyltransferase
WSCs: Water-soluble carbohydrates
ROS: Reactive oxygen species
1-SST: Sucrose-sucrose 1-fructosyltransferase
1-FFT: Fructan-fructan 1-fructosyltransferase
6-SFT: Sucrose-fructan 6-fructosyltransferase
6G-FFT: Fructan-fructan 6G-fructosyltransferase
CWIs: Cell wall invertases
FEHs: Fructan exohydrolases
DAVID: Database for Annotation, Visualization, and Integrated Discovery
S_TKc: Serine-threonine protein kinase catalytic domain
PP2Cc: Serine-threonine phosphatases, family 2C, catalytic domain
HSF: Heat shock factor domain
FH: Formin homology domain
GO: Gene Ontology annotation
PCA: Principle component analysis
KEGG: Kyoto Encyclopedia of Genes and Genomes
LpFTL: *Lolium perenne* FT-like protein
GAPDH: Glyceraldehyde-3-phosphate dehydrogenase
UGPase: UDP-glucose pyrophosphorylase
SCAMP6: Secretory carrier-associated membrane protein
