# Supplementary figures and images for: Changes in Lolium perenne transcriptome during cold acclimation in two genotypes adapted to different climatic conditions
Source: BMC Plant Biol. 2015 Oct 17;15:250. doi: 10.1186/s12870-015-0643-x (PMC4609083; doi:10.1186/s12870-015-0643-x)

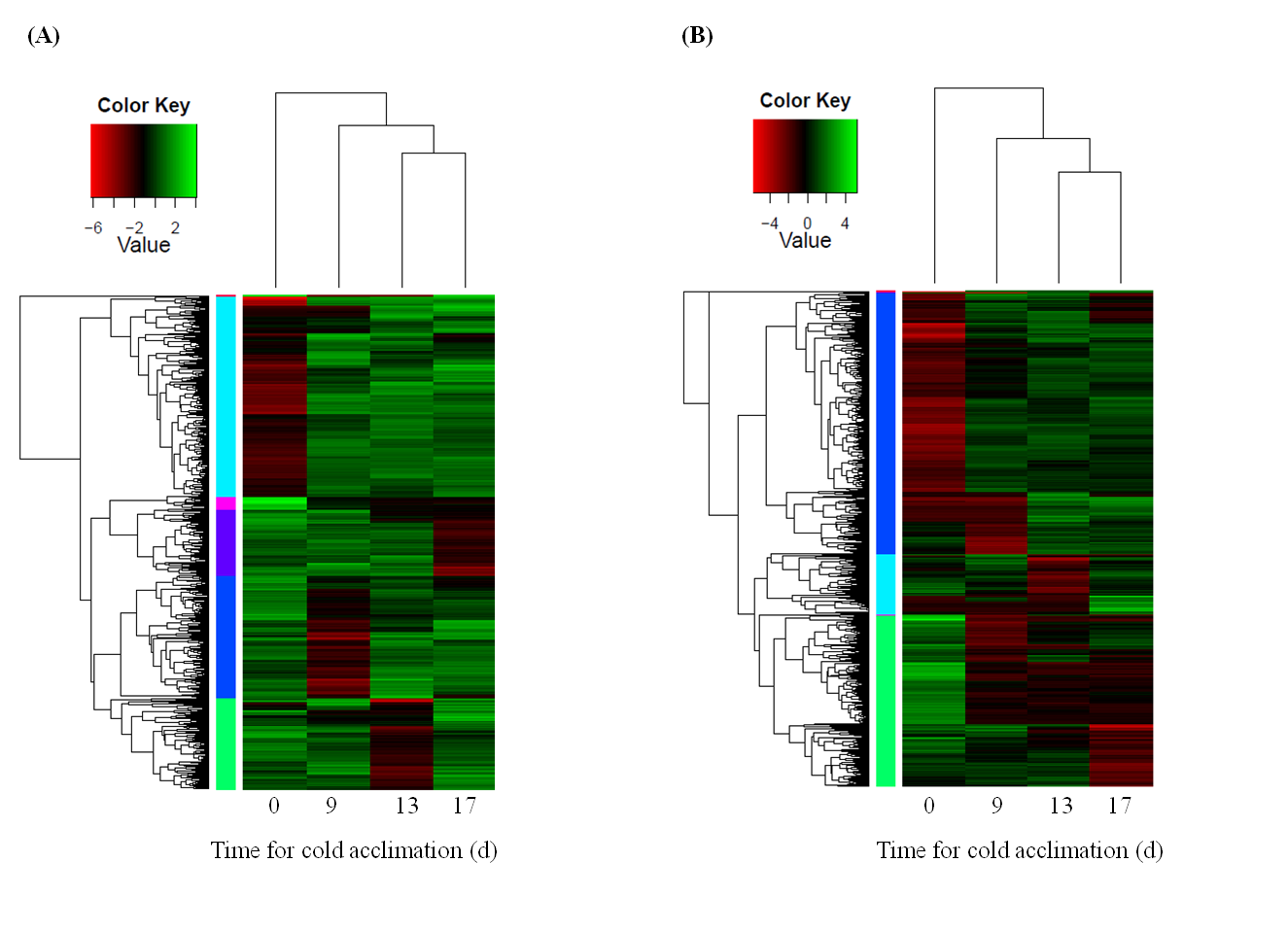

Supplement: Additional file 1: — Heat maps derived from hierarchical clustering of transcripts differentially expressed in ‘Veyo’ and ‘Falster’ during cold acclimation. Expression patterns across d 0, 9, 13, and 17 of cold acclimation in (A) ‘Veyo’ and (B) ‘Falster’. Green represents up-regulation, and red represents down-regulation. (TIFF 494 kb) [file 12870_2015_643_MOESM1_ESM.tif]

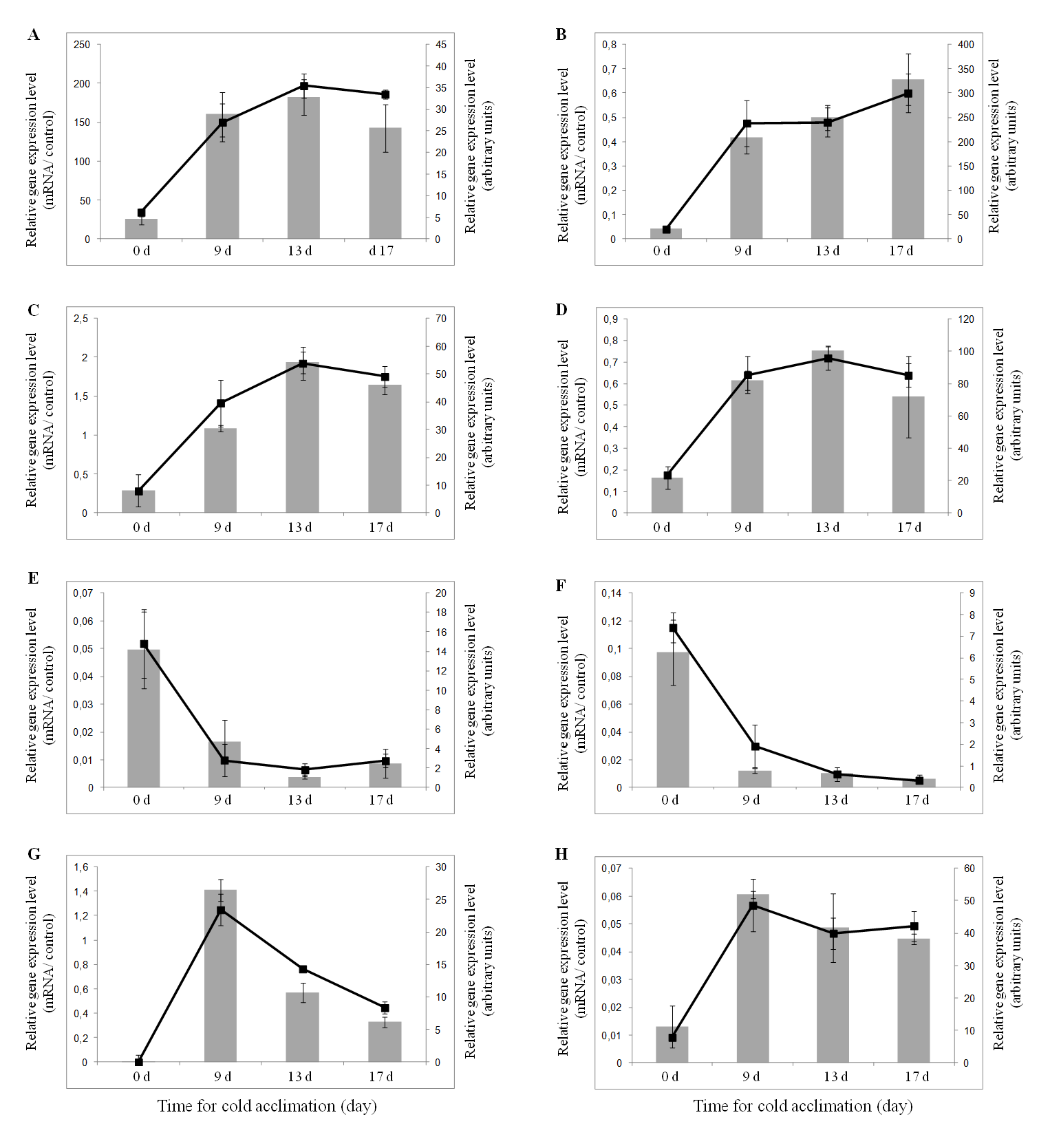

Supplement: Additional file 6: — Validation of RNA-Seq results using quantitative RT-PCR. The expression analysis of selected fructan-related genes differentially expressed during cold acclimation in perennial ryegrass are shown. Plants were at 20 °C on the d 0 and at 7 °C on the d 9, 13 and 17. The normalized transcript levels obtained in the RNA-Seq experiment (lines, right scale) and quantitative RT-PCR results (solid bars, left scale) are shown. Expression of (A) NGB_70583_c0_seq6, (B) NGB_67546_c0_seq4, and (C) NGB_52509_c0_seq1 transcripts from ecotype ‘Falster’, homologues to FTs. Expression of (D) NGB_56538_c0_seq2, and (E) NGB_43481_c0_seq3 transcripts from ecotype ‘Falster’, homologues to Lp-FEH and LpFTL genes respectively. Expression of (F) Veyo_57878_c1_seq2, (G) Veyo_65000_c0_seq1, and (H) Veyo_72656_c0_seq1 transcripts from variety ‘Veyo’, homologues to LpFTL, LpCWI-1 and LpCWI-2 genes respectively. Quantitative RT-PCR data were normalized by geometric averaging of elongation factor 1-alpha (LpEF1a), actin (LpACT11) and eukaryotic initiation factor 4A (LpeIF4a) internal control genes. Data represent mean ± SE obtained from three biological replicates of the analysis. (TIFF 439 kb) [file 12870_2015_643_MOESM6_ESM.tif]
