# Supplementary material for: Changes in Lolium perenne transcriptome during cold acclimation in two genotypes adapted to different climatic conditions
Source: BMC Plant Biol. 2015 Oct 17;15:250. doi: 10.1186/s12870-015-0643-x (PMC4609083; doi:10.1186/s12870-015-0643-x)
Supplement: Additional file 16: — PCR primers used to amplify the template DNA for absolute quantification of gene expression using quantitative RT-PCR. Forward and reverse primers used to amplify the template DNA for the expression analysis of the genes encode fructosyltransferase-like (LpFTL), and cell wall invertases (LpCWI-1 and LpCWI-2). (DOCX 20 kb) [file 12870_2015_643_MOESM16_ESM.docx]

**Additional file 16.** PCR primers used to amplify the template DNA for absolute quantification of gene expression using quantitative RT-PCR. Forward and reverse primers used to amplify the template DNA for the expression analysis of the genes encode fructosyltransferase-like (LpFTL), and cell wall invertases (LpCWI-1 and LpCWI-2).

| Gene | PCR primers (5’-3’) | |
| --- | --- | --- |
| *LpFTL* | Forward | CGATGGACTTACCTCGCTTG |
|  | Reverse | TTGCCAAGGTCGATGGTATT |
| *LpCWI-1* | Forward | TAGATCGATCGGTCGGTGGT |
|  | Reverse | GGTCAGCTTGGACACCTTGA |
| *LpCWI-2* | Forward | GTCCAAGCTGGAGGAGAGG |
|  | Reverse | TGGCTGTGTCACATGATTGAG |
